# Use of Limited Femorotomy as an Alternative to Extensive Trochanteric Osteotomy for Cementless Femoral Prosthesis Revision

**DOI:** 10.1016/j.artd.2025.101640

**Published:** 2025-02-25

**Authors:** Thomas Aubert, Aurelien Hallé, Florian Kruse, Simon Marmor, Luc Lhotellier, Wilfrid Graff

**Affiliations:** Orthopedic Department, Diaconesses Croix Saint Simon Hospital, Paris, France

**Keywords:** Total hip arthroplasty, Limited femorotomy, Cementless stem revision

## Abstract

**Background:**

Cementless stem extraction during hip arthroplasty revision can be challenging and sometimes requires a femoral opening to be performed with limited posterior femorotomy techniques been described. The study objective was to assess the efficacy of these techniques and the perioperative and postoperative complication rates.

**Methods:**

This study included 224 patients who underwent cementless stem revision. Stem extraction followed the same sequence: an initial endomedullary extraction attempt, followed by suspended posterior unicortical vertical diaphyseal femoral osteotomy. Metaphyseal extension of the osteotomy and lateral-distal cortical extension at the stem tip were performed if the procedure failed, followed by extended trochanteric osteotomy (ETO). The incidence rates of perioperative fracture, reimplanted stem type (standard or revision), postoperative subsidence, and fracture were analyzed.

**Results:**

Femoral opening was required in 15.6% of patients; 75% underwent limited femorotomy (28 patients, 75% suspended, and 25% extended), and 25% (7 patients) underwent ETO. Endomedullary extraction was performed in 84.4% (189) of the patients. The perioperative fracture rates were 16.9%, 0%, and 14.3% in the endomedullary, limited femorotomy, and ETO groups, respectively (*P* = .032). The standard stem utilization rates were 94.9%, 82.1%, 58.6%, and 28.6% (*P* < .001) for the endomedullary, limited femorotomy, perioperative fracture, and ETO groups, respectively. Postoperatively, the subsidence rates were 7.5%, 0%, and 28.6% (*P* = .042), and the fracture rates were 4.3%, 3.6%, and 0% (*P* > .999) in the endomedullary, limited osteotomy, and ETO groups, respectively.

**Conclusions:**

Limited femorotomy techniques are reliable methods for extracting cementless stems, when necessary, with a reduced risk of fracture. Postoperatively, these patients appear to have comparable stem subsidence and a low risk of fracture.

## Introduction

The number of hip prostheses implanted each year is increasing [1], with cementless femoral stems gaining popularity [2] owing to their potential longevity and potentially reduced incidence of postoperative fractures or revisions [[3], [4], [5]]. However, the incidence rate of revision total hip arthroplasty (THA) is predicted to increase [6]. The reasons for implant removal vary and include infections, dislocations, length or offset issues, and osteolysis [7,8]. However, the removal of a cementless stem can be challenging, and numerous techniques for fixed stem extraction have been described [[9], [10], [11], [12], [13]]. These methods carry the risk of intraoperative fractures and may necessitate femoral opening, resulting in the use of revision prostheses, contraindication of weight-bearing, the risk of nonunion, and diminished functional outcomes [[14], [15], [16]].

A technique for femoral implant removal using posterior slot femorotomy has been described [13,17,18], allowing partial femoral opening, minimal osteosynthesis, and promising functional results at 5 years. This technique is based on a posterior unicortical diaphyseal saw cut, avoiding metaphyseal extension, to increase femoral elasticity and facilitate the breaking of bridges between the femoral stem and bone. If this proves insufficient, posterior metaphyseal extension and anterior terminal curvature with the saw can be performed to facilitate stem extraction. However, to our knowledge, no study has analyzed the efficacy and safety of limited femorotomy in a consecutive cohort of patients who underwent cementless femoral stem removal. The aim of this study was to analyze the rates of complications associated with limited femorotomy and the need for transition to extended trochanteric osteotomy (ETO) compared with those associated with the endomedullary technique. Additionally, we aimed to determine the rates of intraoperative fractures and the use of revision prostheses. Finally, we analyzed the rates of subsidence and postoperative fractures, the need for prosthetic revisions for femoral mechanical causes, and the overall reasons for revision.

## Material and methods

### Study design and participants

The cohort comprised 963 retrospectively enrolled consecutive patients who underwent hip revision surgery performed by 11 senior surgeons at the same hospital between January 1, 2012, and January 1, 2023. Patients with cemented stems, a history of unipolar cup revisions, periprosthetic fractures, or long locked modular stems for femoral deficiency reconstruction (n = 761) were excluded. Clinical data for 224 patients were collected, anonymized, and retrospectively entered at least 3 months postoperatively. The causes of revisions were infection (168 patients, 75%), loosening (27 patients, 12%), acetabular loosening with incompatibility between the old stem and the new cup (19 patients, 8.4%), subsidence (5 patients, 2.2%), instability (2 patients, 0.9%), ceramic fracture (1 patient, 0.5%), pseudotumour (1 patient, 0.5%), and osteolysis (1 patient, 0.5%). The mean patient age was 66 years (range: 22-91) and the mean body mass index was 27.4 kg/m^2^ (range: 14-38); 109 men (54.0%) and 93 women (46.0%) were included in the study. Table 1 provides the detailed characteristics of the cohort. The stems were short quadrangular (Meije, Corin, or Corail Depuy), anatomical (SPS, Symbios), long quadrangular (Corail Revision Stem), or custom-made (Symbios). Clinical and radiological data were assessed twice by 2 different examiners who were blinded to the outcome. A total of 98.6% of the revisions were performed with a posterior approach, and 1.4% were performed with an anterior approach (which did not require femorotomy) via a chisel, pins, or a specific or universal extractor system. This study was approved by the local ethics committee, and the patients provided informed consent.

**Table 1:** Characteristics of the study population.

Baseline characteristics	Population, n = 224
Background	
Age (y), mean (ranges)	66.2 (22/91)
Height (mm), mean (range)	172 (143/201)
Weight (kg), mean (range)	81.2 (4/135)
BMI (kg/m^2^), mean (range)	27.4 (13.9/37.5)
Implants extracted	
Standard quadrangular stem n (%)	192 (85.7%)
Anatomic, n. (%)	29 (12.9%)
Quadrangular long, n (%)	2 (0.9%)
Customed stem, n (%)	1 (0.4%)

BMI, body mass index.

### Limited femorotomy and complete femorotomy

**Figure 1:**
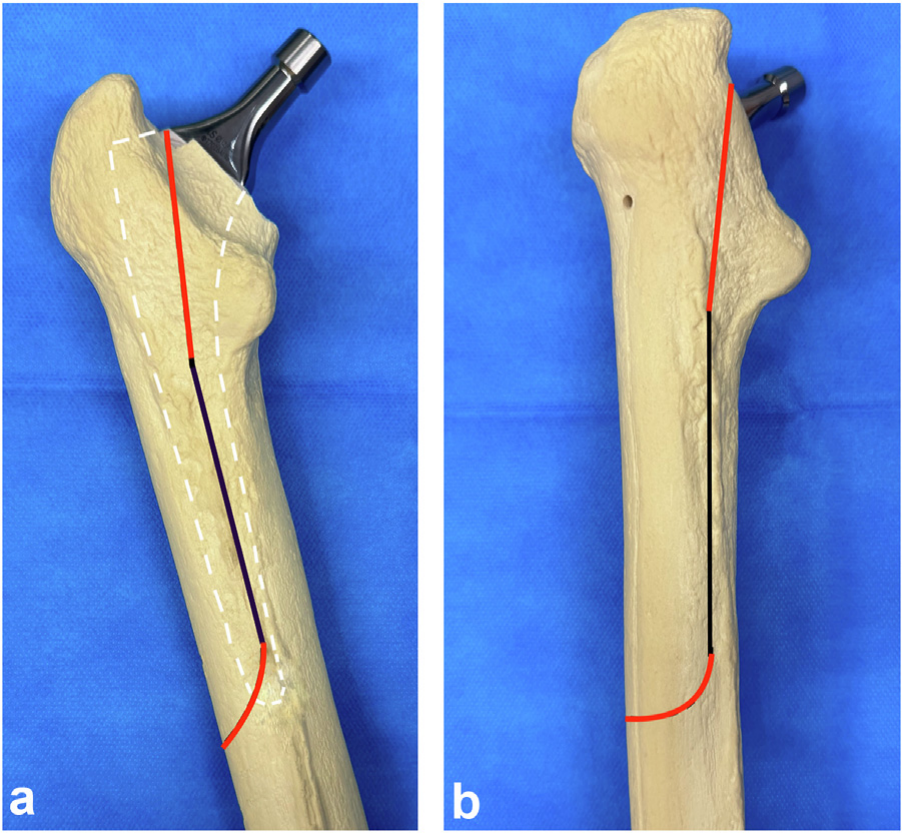
Drawing of limited femorotomy lines. (a) Posterior view of the femur. (b) Three-quarter view of the femur. The black line represents the slot for the limited suspended femorotomy, and the red line represents the proximal and laterodistal extension of the slot for the extended limited posterior femorotomy.

**Figure 2:**
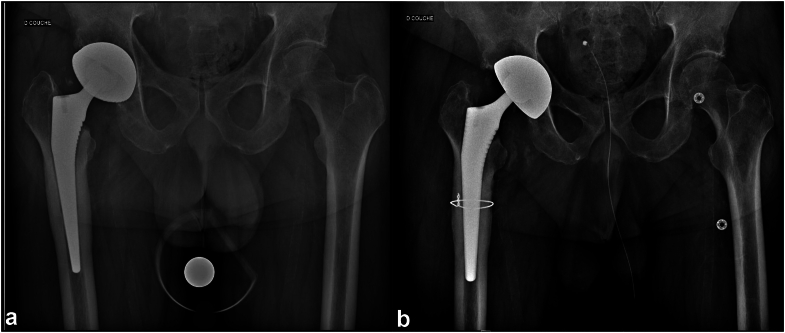
Suspended limited femorotomy. (a) Preoperative radiograph of a patient with right prosthetic hip infection. (b) Postoperative radiograph of a stem extraction with limited suspended femorotomy and reimplantation of a standard stem with 1 wire.

**Figure 3:**
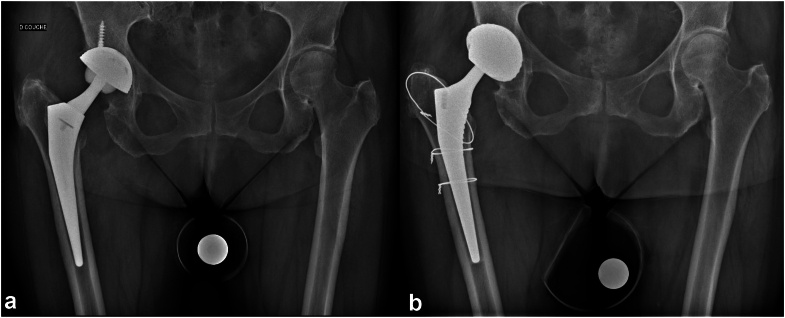
Extended limited femorotomy. (a) Preoperative radiograph of a patient with right prosthetic hip infection. (b) Postoperative radiograph of a stem extraction with limited extended femorotomy and reimplantation of a standard stem with 3 wires.

Limited femorotomy or ETO procedures were employed only if it was impossible to extract the stem with endomedullary procedures. For each surgery, the same sequence was used. An attempt for endomedullary extraction of the femoral stem was made. Chisels and adaptable osteotome handles and blades were used. A universal extractor was subsequently employed to remove the stem. This step is repeated to achieve stem removal. In cases of failure, suspended limited femorotomy was performed via a posterior unicortical diaphyseal saw cut, following the posterior border of the vastus lateralis muscle without metaphyseal extension, starting below the lesser trochanter and ending at the level of the stem's tail. (Fig. 1a). The femorotomy slot was operated via a chisel to manipulate the elasticity of the femur and facilitate the breaking of bridges between the femoral stem and the bone, followed by an extraction attempt via an extractor. Synthesis was performed with one or 2 circumferential wires according to the surgeon's habits (Fig. 2). In cases of failure, extended limited femorotomy was performed by extending the cut into the posterior metaphysis while sparing the greater trochanter and extending laterodistally along the length of the stem with an anterior terminal curvature to minimize the risk of fracture. (Fig. 1b). If the limited suspended femorotomy involves only a diaphyseal femoral opening, its extended version includes an extension of the saw line into the proximal metaphysis and distally onto the anterior cortex. The goal was to facilitate the breaking of the remaining bridges distal to the stem and, through proximal extension, increase femoral elasticity. Osteosynthesis was performed via 3 circumferential wires (Fig. 3). Finally, if this procedure was unsuccessful, classic ETO with a femur opening was performed via an anterior saw cut parallel to the posterior femorotomy cut to open the femur without damaging the greater trochanter and to remove the stem. [14].

### Outcome

The outcome of interest was whether limited femorotomy or ETO was necessary and whether fracture occurred, as identified on postoperative radiograph and in surgical reports during the procedure. The types of stems used for reimplantation, including standard stems (tapered stems with or without collars) and revision stems (with or without screws), were analyzed. At the last follow-up, we investigated the rate of subsidence, defined as vertical migration >3 mm between the tip of the greater trochanter and shoulder of the prosthesis, and the postoperative fracture rate on sequential radiographs during the immediate postoperative period; at days 1, 3, and 1 year, using MediCAD 2D (Hectec GmbH, Ltd., Germany); and the need for reintervention for mechanical reasons.

We classified patients into 3 groups for comparison: patients for whom limited femorotomy was necessary (including suspended and extended femorotomy) (28 patients), those for whom ETO was necessary (7 patients), and those for whom neither was necessary (189 patients).

### Data analyses

Continuous variables are described using means and interquartile ranges. We compared means and proportions between groups via Student’s t tests, analyses of variances (Mann–Whitney tests), or chi-square tests (or Fisher’s exact tests, if appropriate). All analyses were performed via R (version 4.0.0, R Foundation for Statistical Computing, Vienna, Austria; URL: https://www.R-project.org/). *P* < .05 was considered to indicate statistical significance, and all tests were 2-tailed.

## Results

### Intraoperative extraction technique used

The cumulative number of limited femorotomies was 28 (12.5%), including 21 suspended limited femorotomies (75%) and 7 extended limited femorotomies (25%). There were 7 (3.1%) patients who underwent ETO, and 189 (84.4%) patients underwent extractions without femorotomy (Table 2).

**Table 2:** Intraoperative and postoperative characteristics of the study cohort.

Intraoperative and postoperative characteristics	Endomedullary removal n = 189 (84.4%)	Limited femorotomy n = 28 (12.5%)	ETO, n = 7 (3.1%)	*P* value
Intraoperative characteristics				
Fracture, n (%)	32 (16.9)	0 (0)	1 (14.3)	.**032**
Standard stem implanted, n (%)	169 (89.4)	5 (17.8)	5 (71.5)	**<**.**001**
Collared stem implanted, n (%)	65 (34.4)	11 (39.3)	6 (85.7)	.**025**
Postoperative complications				
Subsidence, n (%)	14 (7.5)	0 (0)	2 (28.5)	.**042**
Fracture, n (%)	8 (4.3)	1 (3.5)	0 (0/0)	>.999
Dislocation, n (%)	11 (5.9)	0 (0)	0 (0)	.559
Rerevision for mechanical reasons, n (%)	3 (1.6)	1 (3.6)	1 (14.3%)	.084

Bold values denote statistical significance at the *P* < .05 level.

**Figure 4:**
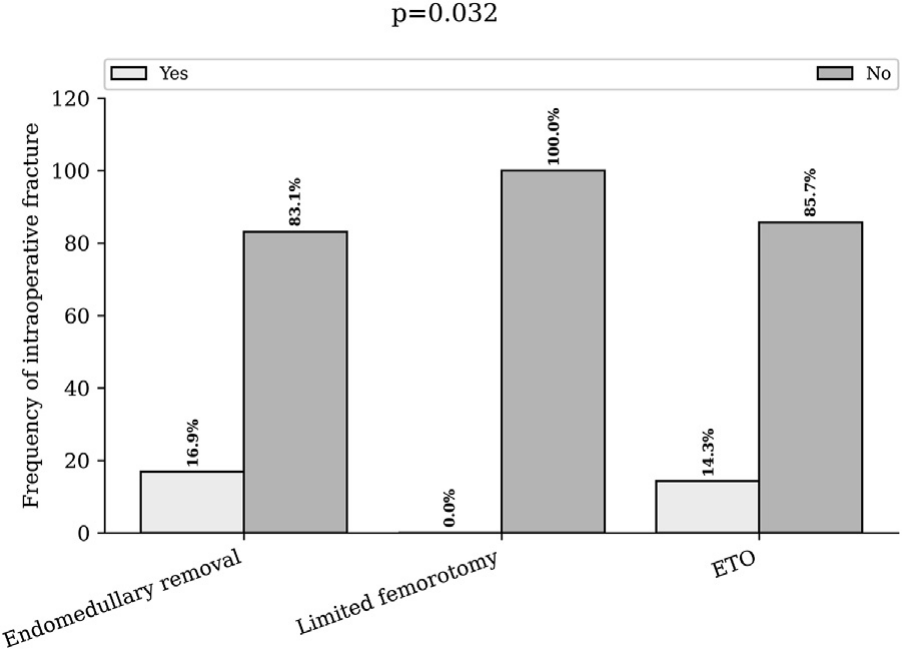
Rate of intraoperative fractures according to the extraction technique used. Bar chart of the intraoperative fracture rate for endomedullary extraction, limited femorotomy, and extended trochanteric osteotomy. ETO, extended trochanteric osteotomy.

The rate of intraoperative fracture was 16.9% (32) in the endomedullary group, 0% in the limited femorotomy group, and 14.3% (1) in the ETO group (*P* = .032) (Fig. 4).

**Figure 5:**
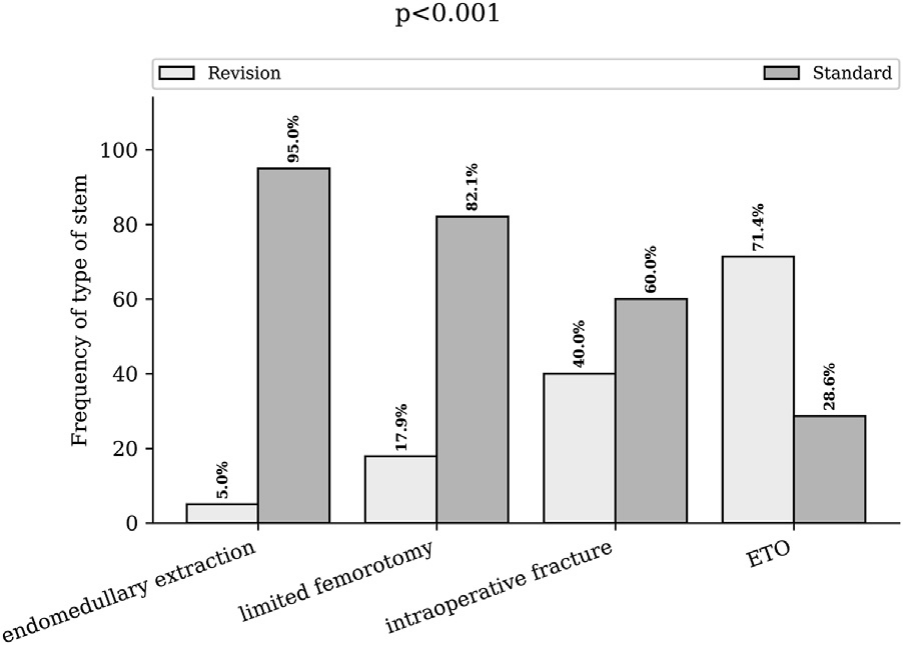
Type of stem reimplanted according to the extraction technique used. Bar chart of the stem rate (standard or revision stems) for endomedullary extraction, limited femorotomy, intraoperative fractures and extended trochanteric osteotomy. ETO, extended trochanteric osteotomy.

All the stems used in the revision in our series were cementless stems. The rate of use of a standard stem was 94.9% in patients who underwent endomedullary extraction without fracture, 82.1% in patients who underwent limited femorotomy, 60% in patients with intraoperative fracture, and 28.6% in patients who underwent ETO (*P* < .001) (Fig. 5). When intraoperative fractures were integrated into the different groups, the rate of resorting to a standard stem in the intramedullary group was 89.4% (169), which was unchanged for the limited femorotomy and ETO groups (*P* < 0,01).

### Postoperative complications

**Figure 6:**
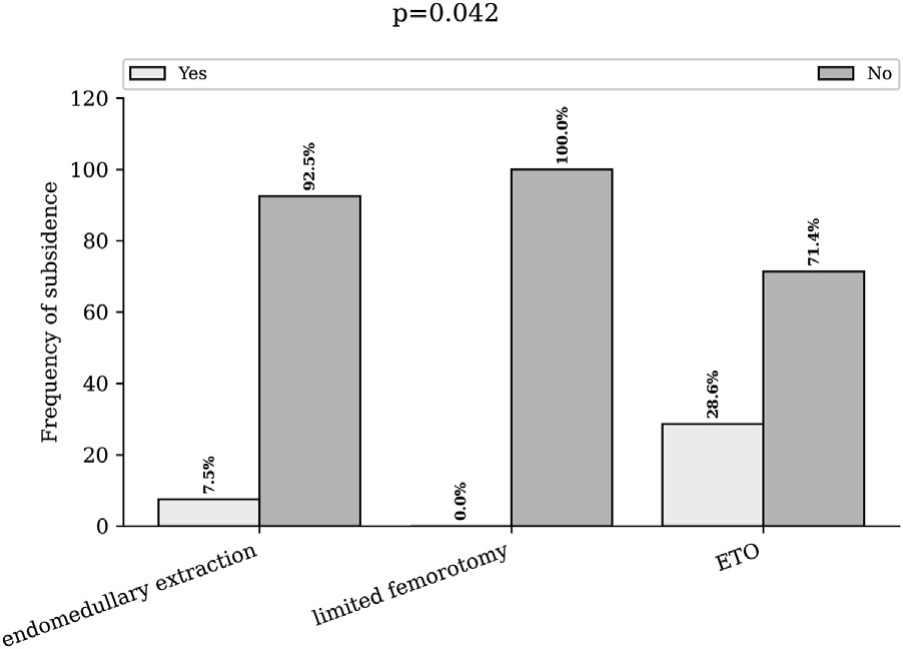
Rate of subsidence according to the extraction technique used. Bar chart of the subsidence rate (more than 3 mm) for endomedullary extraction, limited femorotomy, and extended trochanteric osteotomy. ETO, extended trochanteric osteotomy.

Postoperatively, the subsidence rate was 7.5% in the endomedullary removal group, 0% in the limited femorotomy group, and 28.6% in the ETO group (*P* = .042) (Fig. 6).

**Figure 7:**
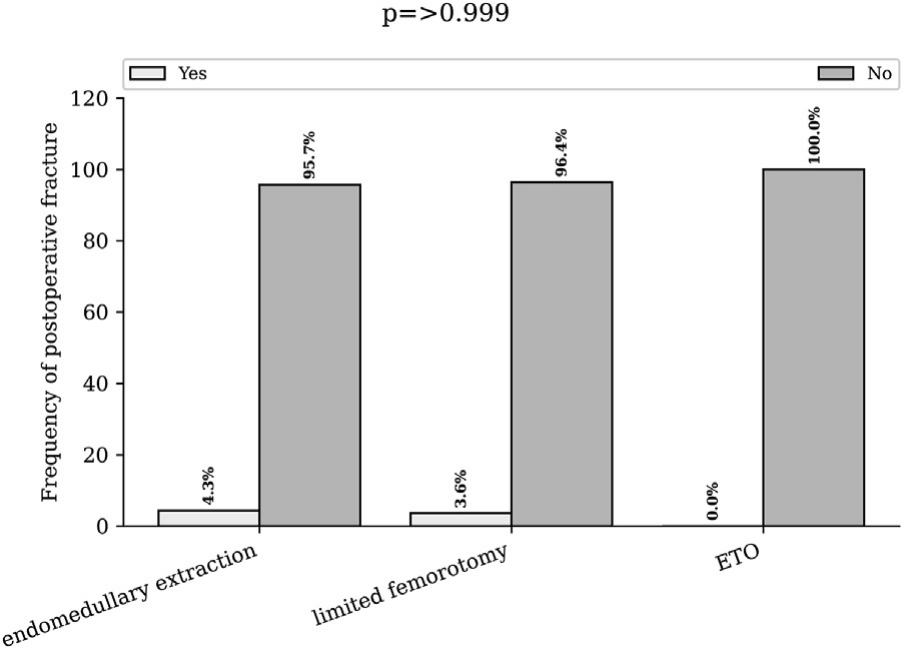
Rate of subsidence according to the extraction technique used. Bar chart of the postoperative fracture rate (more than 3 mm) for endomedullary extraction, limited femorotomy, and extended trochanteric osteotomy. ETO, extended trochanteric osteotomy.

The postoperative fracture rate was 4.3% in the endomedullary group, 3.5% in the limited femorotomy group, and 0% in the complete femorotomy group (*P* > .999) (Fig. 7).

**Figure 8:**
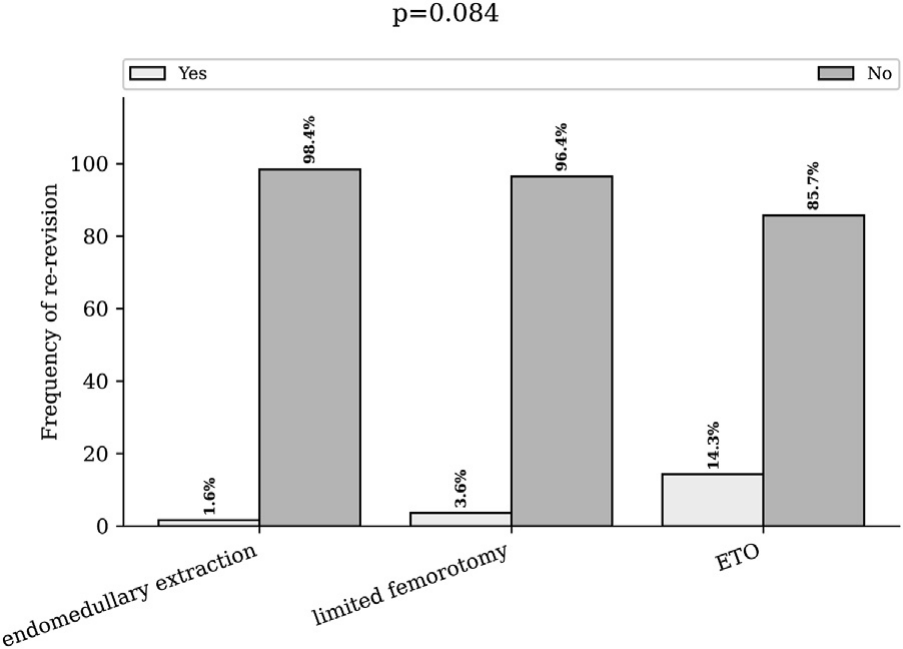
Rate of rerevision according to the extraction technique used. Bar chart of the rerevision rate due to mechanical reasons involving the stem for endomedullary extraction, limited femorotomy, and extended trochanteric osteotomy. ETO, extended trochanteric osteotomy.

The percentage of patients who underwent rerevision for mechanical reasons related to the stem was 1,6% of the patients in the endomedullary removal group, 3.6% in the limited femorotomy group and 14.3% in the ETO group (*P* = .084) (Fig. 8).

The dislocation rate was 5.9% in the endomedullary group, 0% in the limited femorotomy group, and 0% in the complete femorotomy group (*P* = .559).

## Discussion

The extraction of a cementless stem can be difficult and may require a complete opening of the femur [13,14]. The analysis of our consecutive series highlighted the need for a femoral opening in nearly 1 of every 6 patients. It appears that performing limited femorotomy when necessary allows for the removal of the stem without the need for ETO in 75% of patients, with suspended femorotomy used for the majority. While endomedullary removal was possible in the vast majority of patients, the incidence of intraoperative fractures was significant, occurring in nearly 1 out of every 6 patients. Both fractures and complete femorotomy increase the need for revision implants in the majority of patients, potentially resulting in a prolonged operative time and increased infection risk [19]. Conversely, the use of limited femorotomy allowed for the placement of a standard stem with circumferential synthesis and weight-bearing support for 6 weeks. This slot femorotomy technique has been reported to yield good functional results [17,20], although comparisons with other stem extraction techniques are lacking. Although this posterior slot femorotomy technique was described in a 5-year analysis of 15 patients, to our knowledge, no intermediate step has been reported before complete femorotomy. Only a more recent series described a similar technique with metaphyseal extension only, achieving a success rate of 60% in an analysis of 10 patients for whom stem revision was performed [18]. The use of an extended limited femorotomy allowed for simple extension of the proximal and lateral/distal osteotomy lines, increasing bone flexibility and enabling stem removal with a partial femoral opening. This allowed for limited synthesis with 3 circumferential wires and the use of standard stems compared with the reference technique of ETO, which requires more extensive syntheses and reconstruction stems [14,15].

Postoperative subsidence analysis did not reveal an increased risk with the use of this technique, with a subsidence rate of 7% for endomedullary extraction compared with 0% for limited femorotomy. This difference can be explained by bone weakening during endomedullary extraction, resulting in less retention of the reimplanted stem. Furthermore, the risk of postoperative fracture was comparable between the 2 groups. There were no differences in the number of revisions performed for mechanical reasons. However, out of the 7 patients who underwent complete femorotomy, 2 patients required wire removal due to discomfort, particularly with trochanteric wires, which was not the case for patients who underwent limited femorotomy. Therefore, the use of an opening technique does not appear to increase the risk of postoperative mechanical failure [14,15]. Although the fracture rate is high for stem removal [21], performing femoral opening decreases this risk. However, it seems necessary to predict the risk of fracture, and preoperative analysis of factors contributing to difficult cementless stem extraction could help to identify these patients and facilitate the safe execution of limited femorotomy at the beginning of surgery [22].

Studies analyzing a wide range of ETO techniques have revealed that the rate of fracture of the diaphyseal fragment during ETO is 0.5%, and postoperative fracture of the greater trochanter occurs in 7% of hips [14,15]. An analysis of a consecutive series of patients who underwent ETO with a single modular femoral stem revealed a subsidence rate of 8.3%, with an overall midterm complication rate of 33.3% and a revision rate of 13.9%, which were relatively high [23]. Furthermore, a systematic review revealed moderate-quality evidence that the use of ETO in patients without sepsis undergoing single-stage revision THA is safe and effective, with a rate of ETO nonunion of 7% and a rate of subsidence involving >5 mm migration of 7% [15]. This finding demonstrates the importance of the use of limited femoral osteotomy to avoid these risks of complications.

Our analysis has several limitations. First, this was a retrospective analysis, and a prospective study would be desirable. Endofemoral removal of the stem was performed via chisels and a universal extractor; however, techniques involving pins [10] and extraction systems [9,24] have been described with good results and could reduce the need for osteotomy. Furthermore, most of the removed stems were tapered straight with a quadrangular cross-section, with a smaller portion of stems being short or anatomical stems. However, a study describing this technique focusing on anatomical stems with a partial coating [17] showed that this technique is effective, and our study confirmed its effectiveness with quadrangular stems. The decision to perform femorotomy was made at the discretion of the operator without a time limit. It would be interesting to conduct a study analyzing the duration of extraction attempts before operator-indexed femorotomy is performed. There are other femoral approaches for prosthesis revision that we did not use, such as trochanteric slide osteotomy, which involves detaching the greater trochanter with abductor insertion and is rather useful for retaining abductors and acetabular exposure, or the transfemoral approach, which is more anterior-based than posterior-based ETO and is rather useful for anterior-based approaches and anterior femoral remodeling [25].

Furthermore, the use of implants relies on the discretion of surgeons, depending on the condition of the bone stock before replacement, with a preference for maximizing the use of cementless standard stems. We used standard quadrangular stems in the majority of the revisions, which yielded the same clinical results as distal fixating modular stems, with fewer complications and fewer stem revisions using cementless conical stems [26]. We only used tapered stems, but both achieved satisfactory midterm clinical results in revision THA patients. Compared with cylindrical stems, tapered stems seem to result in better bone recovery of the proximal femur, a lower incidence of intraoperative fractures, and a lower postoperative thigh pain rate [27]. We used long tapered cementless stems made of titanium and fully coated with hydroxyapatite in femurs with bone defects, which showed excellent clinical and radiographic outcomes [28,29], and demonstrated that for moderate femoral deficiencies, a monoblock, HA-coated titanium stem is a viable option for revision hip arthroplasty and will enable proximal bone regeneration in some patients. However, we acknowledge that excellent results can be achieved with different stem designs [30,31]. Even though we used only cementless stems for revision, the use of cemented standard or long stems has shown very good outcomes in terms of overall survival [32]. A greater risk of dislocation with cementless stems in the first 3 years was shown, with a rate of 4.9% in our series; however, only 1.3% of the patients required revision for instability. On the other hand, cemented stems have a greater rate of rerevision for aseptic loosening [32,33]. Finally, we reserved the use of interlocking cementless long stems in cases of severe bone defects and/or unstable stems to provide better axial and rotational stability, which provided satisfactory clinical and radiographic results [[34], [35], [36]]. We did not compare the types of fixation; however, a study revealed that both claw-plate fixation and cable-alone fixation could achieve satisfactory outcomes, even if claw-plate fixation is superior to cable-alone fixation in terms of biomechanical reconstruction and gait improvement [37]. We did not use a prophylactic wire before stem removal, which could reduce the risk of fracture during extraction [13]. The use of stems with collars varies among surgeons, and conclusions about their relevance to the risk of subsidence cannot be drawn. However, a recent study revealed a decrease in the risk of stem fractures impacted with a collar in primary THA patients over 65 years of age [38]. While a slight reduction in the rate of subsidence has been demonstrated, no difference was found in terms of fracture, revision, or aseptic loosening [39]. Their use could limit the subsidence rate in revision procedures in the absence of calcar osteolysis.

Although the infection rate is greater in revision surgery, we did not analyze the postoperative infection rate in our series because nearly 70% of the patients underwent surgery for reasons related to sepsis. The use of limited femorotomy techniques, however, does not seem to increase this risk.

## Conclusions

Limited femorotomy techniques are reliable techniques for cementless stem extraction when necessary, reducing the need for ETO with a decreased risk of fracture and the need for a revision stem. Postoperatively, these patients appear to have comparable stem subsidence and a low risk of fracture. However, it seems necessary to identify patients at risk of difficult extraction or fracture prior to performing this technique.

## Acknowledgments

We thank American Journal Experts (AJE) for language editing.

## Conflicts of interest

Thomas Aubert is consultant for Corin and Depuy Synthes. Luc Lhotellier is consultant for Amplitude and Corin. Wilfrid Graff is consultant for Amplitude. Simon Marmor is consultant for Corin and Depuy Synthes. All other authors declare there are no conflicts of interest.

For full disclosure statements refer to https://doi.org/10.1016/j.artd.2025.101640.

## CRediT authorship contribution statement

**Thomas Aubert:** Writing – review & editing, Writing – original draft, Validation, Methodology, Investigation, Formal analysis, Conceptualization. **Aurelien Hallé:** Validation, Formal analysis, Data curation. **Florian Kruse:** Validation. **Simon Marmor:** Validation. **Luc Lhotellier:** Visualization, Validation. **Wilfrid Graff:** Validation.

## Appendix A Supplementary data

Conflict of Interest Statement for Hallé

Conflict of Interest Statement for Kruse

Conflict of Interest Statement for Lhotellier

Conflict of Interest Statement for Aubert

Conflict of Interest Statement for Graff
