# Supplementary material for: Use of Limited Femorotomy as an Alternative to Extensive Trochanteric Osteotomy for Cementless Femoral Prosthesis Revision
Source: Arthroplast Today. 2025 Feb 25;32:101640. doi: 10.1016/j.artd.2025.101640 (PMC11907459; doi:10.1016/j.artd.2025.101640)
Supplement: Conflict of Interest Statement for Graff [file mmc5.pdf]

INDIVIDUAL CONFLICT OF INTEREST STATEMENT

American Association of Hip and Knee Surgeons

(Adopted from the American Academy of Orthopaedic Surgeons disclosure statement)

The following form **must be filled out completely and submitted by each author (example, 6 authors, 6 forms). All items require a response. If there is no relevant disclosure for a given item, enter "*None*."**

Use of limited femorotomy as an alternative to extensive trochanteric osteotomy for cementless femoral prosthesis revision

---

Manuscript Title

1. Royalties from a company or supplier (The following conflicts were disclosed)

   Amplitude

2. Speakers bureau/paid presentations for a company or supplier (The following conflicts were disclosed)

   No

3A. Paid employee for a company or supplier (The following conflicts were disclosed)

   No

3B. Paid consultant for a company or supplier (The following conflicts were disclosed)

   No

3C. Unpaid consultants for a company or supplier (The following conflicts were disclosed)

   No

4. Stock or stock options in a company or supplier (The following conflicts were disclosed)

   No

5. Research support from a company or supplier as a Principal Investigator (The following conflicts were disclosed)

   No

6. Other financial or material support from a company or supplier (The following conflicts were disclosed)

   No

7. Royalties, financial or material support from publishers (The following conflicts were disclosed)

   No

8. Medical/Orthopaedic publications editorial/governing board (The following conflicts were disclosed)

   No

9. Board member/committee appointments for a society (The following conflicts were disclosed)

   No

Each author must sign AND print or type his/her name, date and submit a separate form

In addition, one BLINDED Conflict of Interest form (no author names used) should be submitted per manuscript with all author disclosures.

GRAFF Wilfrid		06/04/2024
Author Name (Print or Type)	Author Signature	Date
